# Humanitarian strategies for tackling public health crises in conflict zones in Africa

**DOI:** 10.4102/jphia.v15i1.824

**Published:** 2024-11-21

**Authors:** Mehad Nasreldin, Tamrat Shaweno, Nebiyu Dereje, Nicaise Ndembi

**Affiliations:** 1 Africa Centres for Disease Control and Prevention (Africa CDC), Addis Ababa, Ethiopia

## Introduction

In conflict-affected regions of Africa, the convergence of violence, displacement, and disrupted infrastructure creates significant public health challenges. Armed conflicts cause immediate harm, resulting in trauma, injuries, and urgent medical needs. Survivors face both physical wounds and severe mental and psychosocial impacts such as post-traumatic stress disorder (PTSD), depression, and anxiety. Moreover, armed conflicts increase the vulnerability of women and children and increase the risk of sexual and gender-based violence and child malnutrition.^1^ The destruction of healthcare facilities directly exacerbates the crisis by limiting access to essential services and facilitating the spread of infectious diseases. In Sudan, the ongoing armed conflict has severely compromised healthcare services. As of July 2023, less than one-third of hospitals in conflict zones remain operational, with 70% of hospitals out of service because of artillery attacks, forced militarisation, power outages, and shortages of medical supplies and personnel. This disruption has led to the interruption of essential health services, including obstetric care, emergency services, and dialysis, significantly impacting public health.^2^ Additionally, the health workforce struggles because of inadequate training and resources, primarily caused by limited investment in health infrastructure and education.^3^

As the primary focus of care will be shifted towards managing acute cases during armed conflicts, essential healthcare services will be compromised – leaving a big gap for the spread of infectious diseases, maternal and child healthcare services, and other routine healthcare programmes. Consequently, the countries’ progress towards achieving global targets such as the Sustainable Development Goal and Universal Health Coverage will be severely affected.^4,5,6,7^ This editorial explores effective humanitarian strategies to mitigate public health crises in Africa’s conflict zones, focusing on ensuring healthcare continuity and sustainable recovery.

## Strategies for addressing public health crises in conflict zones

Addressing public health challenges during armed conflicts requires a robust and comprehensive framework that can address both the immediate and long-term impacts of the armed conflict on public health. We call upon all the public health community and various stakeholders to implement the following four critical considerations to effectively and sustainably address the impacts of the armed conflict on public health:

- Most importantly, ensuring the continuity of essential healthcare services through a balanced approach is crucial. Emergency care and essential healthcare services must be balanced throughout armed conflict. This can be ensured through the implementation of mobile clinics and telemedicine to increase access to essential healthcare services, particularly in addressing the health needs of the displaced population. The mobile clinics are crucial for delivering essential medical care in areas where healthcare facilities have been destroyed or are inaccessible because of ongoing conflict. On the other hand, telemedicine can bridge healthcare delivery gaps by offering remote consultations and support, ensuring that even in the most remote or dangerous areas, medical advice and care are accessible.
- Building the capacity of the local health workforce, including the community health workers, and sustaining them is critical to effectively mitigate the impacts of armed conflict. Continued monitoring of the active health workforce in the conflict-affected areas and working towards ensuring their safety and security need to be considered. Investing in the training and empowerment of these workers ensures that healthcare services can continue even when external aid is limited.
- Harmonisation of efforts and resources needs to be considered to increase the effectiveness and sustainability of interventions to address both immediate and long-term impacts of armed conflicts on public health. International partners, civil society organisations, and local governments can play pivotal roles in mitigating the impacts of armed conflicts if they harmonise their resources, expertise, and efforts. Researching, measuring and documenting the impacts of armed conflict also require concerted and harmonised efforts of various stakeholders.^8^
- Integration and alignment of interventions that address the impacts of armed conflicts with the priority programmes of the country is pivotal for its effective and sustainable implementation. This approach will increase resource availability, efficiency, and comprehensive building up of the health system.^9,10^

## Conclusion

Addressing public health crises in conflict zones in Africa requires a multifaceted approach that balances immediate care with long-term recovery. The strategies outlined – ranging from mobile clinics and telemedicine to local capacity building and adaptive research – underscore the importance of flexibility, collaboration, and context-specific interventions. By aligning humanitarian efforts with development goals and empowering local communities, it is possible to create sustainable health systems that can withstand the challenges of conflict and ensure the well-being of affected populations.
